# Childhood Cancer in Pakistan

**DOI:** 10.18502/ijph.v49i8.3908

**Published:** 2020-08

**Authors:** Muhammad SOHAIL AFZAL

**Affiliations:** Department of Life Sciences, School of Science, University of Management and Technology (UMT), Lahore, Pakistan

## Dear Editor-in-Chief

Pakistan is a densely populated, low-income country with an estimated population of 21 million (1). Pakistan is endemic for several communicable and non-communicable mainly due to unawareness in general population, unhygienic lifestyle, poor sanitary conditions in populated areas (2). There are very few studies about the incidence and prevalence of cancer in Pakistan. Previously there are only two cancer registries located in Karachi and Lahore cities and recently (in 2015) a National Cancer Registry was established in Pakistan Health Research Council (PHRC) Islamabad (3). The age standardized ration for cancer in females is slightly higher (172/100000) then males (145/100000). Recently, there are 150,000 new cancer cases in Pakistan and 60%–80% of these patients die (4). About 7000 to 7500 children get cancer every year in Pakistan (5).

Malnutrition and communicable diseases are the main killers of children in Pakistan. Due to better diagnostic facilities, cancer is becoming a serious factor in childhood morbidity and mortality. According to available data, childhood cancer accounts for 10% of all reported cancers in 2017 (5–6). Leukemia (31%) and lymphomas (20%) are the major childhood cancer as per Karachi cancer registry Pakistan (5). Punjab cancer registry data showed that lymphomas (31%) are relatively higher prevalent than leukemia (23%) (6). The available data at newly established PHRC National Cancer Registry is very limited and is not showing the prevalence of childhood cancers (3).

There is a dire need for an accurate surveillance system for cancer incidence and mortality. There are very few populations based cancer registries in the country. In a country with more than 21 million inhabitants, there should be more cancer registries in the country. The data collection system needs to improve and the collected data should be combined at national level. Only when such data are available, this will be of help for policy-makers to make sensible decisions on allocation of precious healthcare resources.
